# Clinical Performance of Analog and Digital 18F-FDG PET/CT in Pediatric Epileptogenic Zone Localization: Preliminary Results

**DOI:** 10.3390/biomedicines13081887

**Published:** 2025-08-03

**Authors:** Oreste Bagni, Roberta Danieli, Francesco Bianconi, Barbara Palumbo, Luca Filippi

**Affiliations:** 1 Department of Nuclear Medicine, Santa Maria Goretti Hospital, AUSL Latina, 04100 Latina, Italy; o.bagni@ausl.latina.it; 2 Department of Human Sciences and Promotion of the Quality of Life, University San Raffaele, Via di Val Cannuta 247, 00166 Rome, Italy; roberta.danieli@uniroma5.it; 3 IRCCS San Raffaele Roma, Via della Pisana 235, 00163 Rome, Italy; 4 Department of Engineering, Università degli Studi di Perugia, Via Goffredo Duranti 93, 06125 Perugia, Italy; francesco.bianconi@unipg.it; 5 Section of Nuclear Medicine and Health Physics, Department of Medicine and Surgery, Università degli Studi di Perugia, Piazza Lucio Severi 1, 06132 Perugia, Italy; barbara.palumbo@unipg.it; 6 Department of Biomedicine and Prevention, University of Rome ‘Tor Vergata’, Via Montpellier 1, 00133 Rome, Italy

**Keywords:** neuroimaging, PET/CT, brain, epilepsy, neurological disorders, ^18^F-FDG

## Abstract

**Background**: Despite its central role in pediatric pre-surgical evaluation of drug-resistant focal epilepsy, conventional analog ^18^F-fluorodeoxyglucose (^18^F-FDG) PET/CT (aPET) systems often yield modest epileptogenic zone (EZ) detection rates (~50–60%). Silicon photomultiplier–based digital PET/CT (dPET) promises enhanced image quality, but its performance in pediatric epilepsy remains untested. **Methods**: We retrospectively analyzed 22 children (mean age 11.5 ± 2.6 years) who underwent interictal brain ^18^F-FDG PET/CT: 11 on an analog system (Discovery ST, 2018–2019) and 11 on a digital system (Biograph Vision 450, 2020–2021). Three blinded nuclear medicine physicians independently scored EZ localization and image quality (4-point scale); post-surgical histology and ≥1-year clinical follow-up served as reference. **Results**: The EZ was correctly identified in 8/11 analog scans (72.7%) versus 10/11 digital scans (90.9%). Average image quality was significantly higher with dPET (3.0 ± 0.9 vs. 2.1 ± 0.9; *p* < 0.05), and inter-reader agreement improved from good (ICC = 0.63) to excellent (ICC = 0.91). **Conclusions**: Our preliminary findings suggest that dPET enhances image clarity and reader consistency, potentially improving localization accuracy in pediatric epilepsy presurgical workups.

## 1. Introduction

Interictal ^18^F-fluorodeoxyglucose positron emission tomography/computed tomography (^18^F-FDG PET/CT) plays a crucial role in the presurgical evaluation of drug-resistant focal epilepsy, particularly in pediatric populations, by identifying the epileptogenic zone (EZ) visualized as regions of hypometabolism [1,2]. Despite its widespread use, conventional analog PET (aPET) systems demonstrate only moderate sensitivity in localizing the EZ, with reported detection rates (DRs) around 50–60% in children and adults alike [3,4]. Such limitations may hamper optimal surgical planning and outcomes in pediatric patients, for whom early and accurate localization is especially critical given the potential impact of uncontrolled seizures on neurodevelopment and quality of life.

Pediatric focal epilepsy presents unique challenges. The developing brain exhibits evolving metabolic patterns and structural heterogeneity, which can complicate interpretation of PET findings. These evolving metabolic patterns encompass dynamic changes in cerebral glucose uptake during brain maturation, age-dependent regional variability related to synaptic pruning, and the metabolic demands of progressive myelination. Such factors can alter baseline ^18^F-FDG distribution in children and may confound the delineation of subtle hypometabolic foci [5]. Moreover, children often require sedation or general anesthesia for prolonged imaging, raising concerns about scan duration and radiation exposure. In this context, any improvement in image quality or reduction in acquisition time can have significant clinical benefit [6]. ^18^F-FDG PET/CT complements structural imaging modalities (MRI) by supplying complementary information to structural MRI, particularly in MRI-negative cases, thereby providing critical functional information that can guide electrode placement for intracranial EEG and/or facilitate the identification of the resection margins [7]. However, when the sensitivity of aPET is suboptimal, false negatives may occur, potentially leading to missed EZs and/or incomplete resections.

Recent advances in PET detector technology, notably the advent of digital PET (dPET) systems based on silicon photomultipliers (SiPM), have shown promise in enhancing image quality, contrast recovery, and timing resolution [8]. SiPM-based detectors offer higher photon sensitivity, improved time-of-flight (TOF) performance, and superior spatial resolution compared to traditional photomultiplier tube (PMT) analog systems [9]. In oncological and neurological imaging contexts, dPET has demonstrated better lesion detectability, sharper lesion delineation, and potential for dose reduction or shorter acquisition times [10]. While much of the initial validation of SiPM PET systems has focused on oncology, there is growing interest in their application to neurological disorders, including epilepsy, where subtle metabolic changes must be reliably detected.

In pediatric epilepsy, the potential advantages of dPET include improved signal-to-noise ratio in small brain structures, clearer differentiation of hypometabolic cortex, and enhanced inter-reader agreement. Better image contrast may facilitate detection of focal cortical dysplasia, mild encephalitic changes, or other etiologies underlying seizures that could escape detection with aPET. Furthermore, higher sensitivity could translate into lower injected activity of ^18^F-FDG, thereby reducing radiation burden without sacrificing diagnostic performance, or alternatively maintain dose while shortening scan duration—both favorable in pediatric practice where minimizing anesthesia time and radiation exposure is paramount [11].

Despite these theoretical advantages, data comparing diagnostic performance of dPET versus aPET specifically in pediatric epilepsy remain scarce. Accordingly, we conducted a retrospective preliminary study comparing ^18^F-FDG PET/CT examinations performed on an analog system (aPET) versus a SiPM-based digital system (dPET) in pediatric patients undergoing presurgical evaluation for focal epilepsy. Leveraging post-surgical histology and clinical follow-up as reference standards, we assessed and compared the detection rate of the EZ, image quality scores, and inter-reader agreement between the two systems.

## 2. Materials and Methods

### 2.1. Study Design

This single-center retrospective study included consecutive pediatric patients (aged < 18 years) with a clinical diagnosis of drug-resistant focal epilepsy who were referred to the Nuclear Medicine Section of Santa Maria Goretti Hospital, Latina (Italy), for pre-surgical evaluation with brain ^18^F-FDG PET/CT. The study period spanned from January 2018 to September 2021. Imaging was performed using either an analog (aPET) or digital (dPET) PET/CT system.

Due to logistical limitations, sedation could not be administered to pediatric patients at our facility. Therefore, all patients were screened prior to PET/CT to assess their compliance and ability to undergo the procedure without sedation. Patients for whom adequate cooperation was not expected—due to young age or other factors—were referred to specialized pediatric centers and excluded from the study. Inclusion criteria were (1) unifocal epilepsy, as indicated by the electroencephalogram (EEG) and semiology; (2) availability of complete PET/CT imaging data; (3) availability of a histopathological report; and (4) a clinical follow-up of at least 1 year [12]. Exclusion criteria were (1) unknown EZ and (2) absence of surgical intervention.

### 2.2. PET/CT Acquisition Protocols

All patients underwent brain ^18^F-FDG PET/CT imaging under standardized conditions. In all cases, PET/CT was carried out during the interictal phase (at least 24 h after the most recent seizure). An in-house monitoring was started 24 h before and continued through the PET scan to detect any subclinical or amnestic seizures that could result in inaccurate findings, such as false hypermetabolism or isometabolism [13]. Patients were required to fast for at least 6 h prior to imaging to optimize cerebral ^18^F-FDG uptake and minimize the influence of elevated serum glucose levels. ^18^F-FDG was administered only if blood glucose levels were below 8.8 mmol/L. The administered activity was calculated based on the EANM Dosage Card [14]. During the uptake phase, patients rested in a dimly lit, quiet environment with minimal sensory stimulation. Imaging was then acquired using either an analog (Discovery ST, GE Healthcare, Chicago, Illinois, US) or digital PET/CT system (Biograph Vision 450 Siemens Healthineers, Erlangen, Germany), depending on availability at the time of the exam. Detailed acquisition and reconstruction parameters for each system are provided in Supplementary Materials.

### 2.3. Qualitative Image Analysis

Qualitative visual analysis of all PET/CT scans was independently performed by three experienced nuclear medicine physicians (Luca Filippi, Oreste Bagni, Roberta Danieli), each with more than 10 years of expertise in pediatric neuroimaging. The reviewers were blinded to (a) the type of PET/CT system used (analog or digital); (b) all clinical and demographic data, including seizure semiology and EEG findings; and (c) structural MRI results.

Each reviewer analyzed the scans independently and in random order, with no access to the evaluations of the other observers. Initially, the observers assessed ^18^F-FDG uptake asymmetry using axial and coronal PET images. They then identified the anatomical location of the EZ. EZ detection was considered accurate if both the laterality and the anatomical subregion matched the site of surgical resection. For sensitivity analysis of aPET and dPET, the EZ was deemed accurately classified on PET imaging when at least one of the three independent readers successfully identified its location in concordance with the site of surgical resection and histopathological confirmation.

Image quality for aPET and dPET was assessed using a modified version of the Paldino scoring system [15,16], which semiquantitatively evaluates the detectability and characteristics of hypometabolic regions. The score ranges from 4 to 1: Score 4: clear identification of both laterality and EZ borders; Score 3: clear laterality with slightly obscured borders; Score 2: possible laterality with unclear borders; Score 1: poor detection of both laterality and borders. The scores assigned by each blinded reader were systematically recorded, entered into a centralized database for tabulation, and subsequently compared both across readers—using intraclass correlation coefficients to assess inter-reader agreement—and against the reference standard (histology and follow-up).

### 2.4. Quantitative Image Analysis

Quantitative PET/CT analysis was performed by a fourth nuclear medicine physician (Barbara Palumbo), also blinded to clinical data and acquisition modality. CortexID Suite (GE Healthcare, Chicago, IL, USA), a commercially available software platform for semi-quantitative brain PET analysis, was used. CortexID compares patient scans with an age-matched normative database and generates z-score maps to highlight statistically significant regional metabolic abnormalities (Figure S1) [17]. For each spatially and globally normalized scan, z-scores were computed for the following bilateral regions of interest (ROIs): lateral prefrontal cortex (LPFC), medial prefrontal cortex (MPFC), superior parietal cortex (SPC), inferior parietal cortex (IPC), lateral temporal cortex (LTC), mesial temporal cortex (MTC), lateral occipital cortex (LOC), and precuneus [18]. Negative z-scores indicated relative hypometabolism. Voxel-wise analysis focused on cortical and subcortical regions potentially involved in seizure onset, based on surgical outcomes.

### 2.5. Statistical Analysis

Data are presented as mean ± standard deviation, median, or number (percentage). Statistical analyses were performed using MedCalc software (version 11.3.8.0, MedCalc Software, Mariakerke, Belgium). Fisher’s exact test was applied to examine the differences in detection rate between digital and analog system, while 95% confidence intervals were estimated by Agresti-Coull approximation for binomial proportions [19]. The intraclass correlation coefficient (ICC) was calculated to assess interobserver agreement and was interpreted as follows: excellent (ICC > 0.8), good (ICC > 0.6), moderate (ICC > 0.4), and poor (ICC ≤ 0.4). A two-sample *t*-test was used to compare Paldino and z-scores between aPET and dPET systems. Regression analysis was performed to evaluate the correlation between visual (Paldino) and semiquantitative (CortexID) scores. A *p*-value < 0.05 was considered statistically significant.

### 2.6. Standard of Reference and Post-Surgical Outcome

The reference standard for localization of the EZ was defined as the combination of post-surgical histopathological diagnosis and clinical follow-up of at least 1 year. All 22 patients were evaluated within a multidisciplinary team that integrated all available clinical and diagnostic information, including ictal semiology, electrophysiological data (i.e., EEG findings), metabolic patterns from PET imaging, and structural abnormalities identified on MRI. Based on the comprehensive assessment from this multidisciplinary discussion, individualized surgical plans were developed for each patient. Following surgery, all treated patients were systematically re-evaluated by the same multidisciplinary team during postoperative follow-up. Seizure outcomes were classified according to the Engel classification system, which provides a standardized framework for assessing postsurgical seizure control [20].

## 3. Results

A total of 22 pediatric patients (mean age 11.5 ± 2.6 years, range 7–17 years; 13 males, 9 females) with drug-resistant focal epilepsy (mean epilepsy duration 3.1 ± 1.2 years, range 2–6 years) underwent presurgical brain ^18^F-FDG PET/CT between January 2018 and September 2021. Of these, 11 patients were scanned using aPET, while the remaining 11 were imaged with a dPET. Detection rates for EZ localization were 72.7% (95% CI 42.9–90.8%) with aPET and 90.9% (95% CI 60.1–100.0%) with dPET, although the difference did not reach statistical significance (*p* = 0.5).

Final diagnosis localized the EZ primarily to the temporal lobe: eight cases in the right temporal lobe and seven in the left. Additionally, five patients presented frontal lobe involvement, one patient had combined left frontotemporal localization, and one had an extension into the occipital lobe. Surgical histopathology confirmed hippocampal sclerosis (HS) in 10 patients, focal cortical dysplasia (FCD) in 5, and reactive gliosis in another 5. One patient had a temporal lobe cyst, and another was diagnosed with a WHO grade 2 oligodendroglioma (ODG). After surgery, of the 22 patients, 16 (72.7%) achieved Engel class I seizure freedom at 12-month follow-up (7/11 in aPET; 9/11 in dPET), and 6 (27.3%) were Engel II-III. Patient clinical and demographic data, as well as the results of PET scans and histology are shown in Table 1.

### 3.1. Visual and Quantitative Scoring, Interobserver Agreement

**Figure 1:**
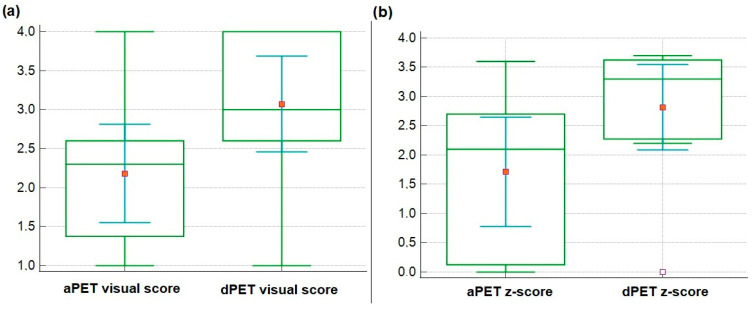
Box-plot diagrams representing the visual (**a**) and z-scores (**b**) obtained with the analog (aPET) and digital (dPET) systems, respectively.

Visual assessment scores were significantly higher for dPET than aPET (mean score: 3.0 ± 0.9 vs. 2.1 ± 0.9; *p* = 0.004), as reported in Table 2. Correspondingly, the z-scores were also significantly higher for dPET (2.8 ± 1.0) than aPET (1.7 ± 1.3; *p* = 0.03). Figure 1 illustrates the distribution of both visual and z-scores across the two imaging systems.

**Figure 2:**
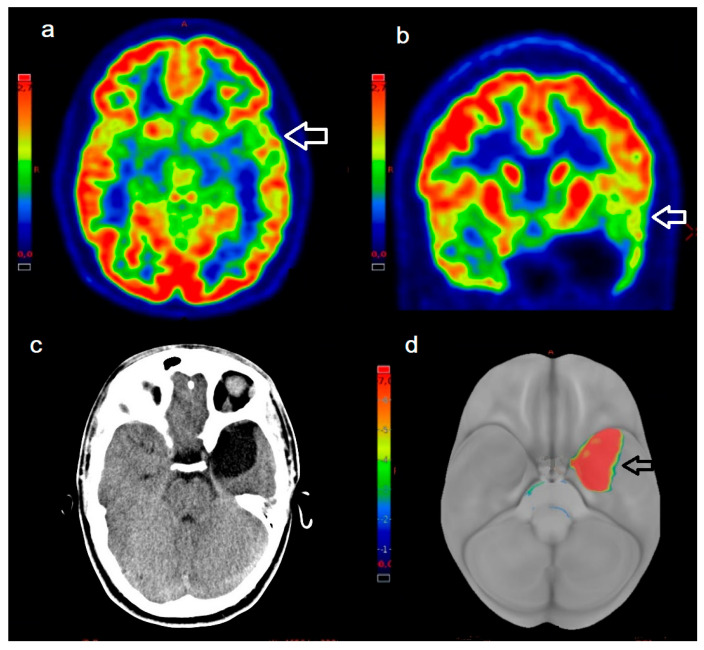
A 13-year-old male patient with a left temporal lobe cyst and epilepsy characterized by absence seizures and clonic–tonic manifestations, submitted to ^18^F-FDG aPET. Axial (**a**) and coronal (**b**) PET images show asymmetric tracer distribution in the left temporal region near the supero-lateral margin of the temporal cyst (white arrows), clearly visualized on the co-registered CT scan (**c**). Qualitative evaluation using the Paldino score yielded a value of 2 (i.e., possible laterality with unclear borders), with full inter-observer agreement. Panel (**d**) shows a 3D volume rendering of the Z-score in the left temporal lobe (−0.6; black arrow). The patient underwent surgical removal of the cyst, which was causing an irritative effect on the adjacent parenchyma, resulting in symptomatic improvement.

**Figure 3:**
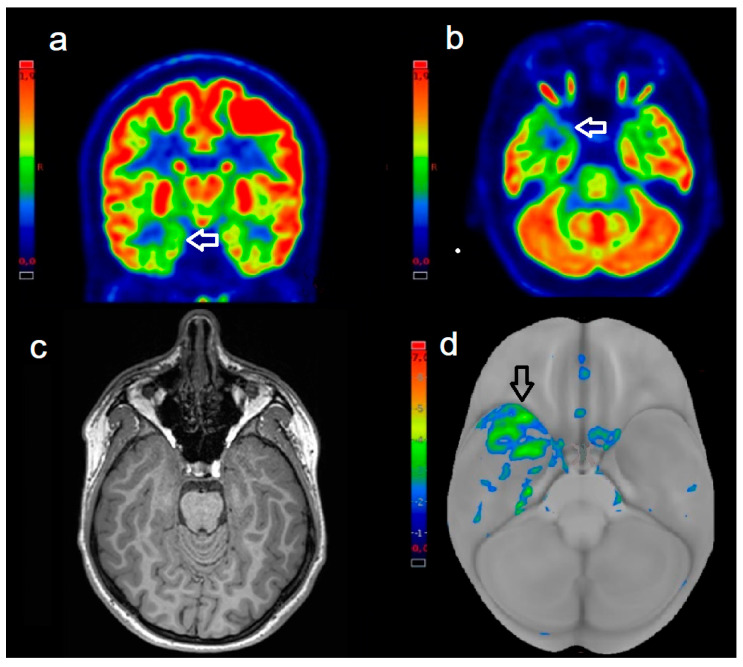
A 13-year-old girl with pharmacoresistant focal epilepsy, submitted to ^18^F-FDG dPET. Coronal (**a**) and axial (**b**) ^18^F-FDG-PET images demonstrate marked cortical hypometabolism in the right mesial temporal region (white arrows). (**c**) Corresponding axial T1-weighted MRI shows no obvious structural abnormality. (**d**) 3D volume rendering of the z-score map (z-score= −3.4) highlights the epileptogenic zone in the right temporal lobe (black arrow). Qualitative assessment using the Paldino score was 4, indicating unambiguous lateralization and clear definition of epileptogenic borders. The patient underwent surgical resection, histopathology confirmed hippocampal sclerosis, and she experienced significant seizure reduction at follow-up.

Interobserver agreement, measured using ICC, was good for aPET (ICC = 0.63) and excellent for dPET (ICC = 0.91). This indicates that the digital system provided more consistent evaluations among readers, especially in delineating the location and extent of abnormal versus normal regions. Representative cases from the aPET and dPET groups are shown in Figure 2 and Figure 3, respectively.

### 3.2. Correlation Between Visual and Z-Scores

**Figure 4:**
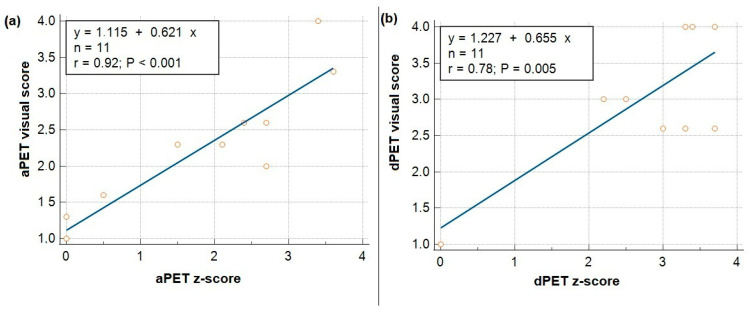
The plots show the relationship between visual and z-scores for analog (**a**) and digital PET (**b**), with each data point representing an individual measurement.

Statistical analysis revealed a strong and statistically significant correlation between visual scores and z-scores for both imaging systems. For aPET, the correlation coefficient was r = 0.92 (*p* < 0.001), and for dPET, r = 0.78 (*p* = 0.005). These relationships are presented in Figure 4.

### 3.3. Correlation Between PET and MRI Findings

In the aPET cohort, 8 patients had MRI-visible lesions: hypometabolism fully overlapped the MRI lesion in 7/8 cases (87.5%) and was discordant in 1/8 (12.5%). Among the 3 MRI-negative aPET patients, in all cases aPET identified a potential EZ.

In the dPET cohort, 7 patients had MRI lesions: dPET concordantly localized hypometabolism in 6/7 cases (85.7%) and was discordant in 1/7 (14.3%). Of the 4 MRI-negative dPET patients, dPET detected an EZ in 4 (100%). These findings illustrate similar MRI–PET concordance rates in MRI-positive and MRI-negative cases for both modalities (i.e., aPET and dPET).

## 4. Discussion

In this preliminary investigation of pediatric presurgical ^18^F-FDG PET/CT, SiPM-based dPET demonstrated several clear advantages over conventional aPET. Although the increase in EZ detection—from 72.7% to 90.9%—did not reach statistical significance, this is compatible with the limited sample size and retrospective design. More striking, however, was the nearly one-point gain in mean subjective image quality scores and the jump in inter-reader reliability from good (ICC = 0.63) to excellent (ICC = 0.91). These findings suggest that the superior count sensitivity, timing resolution, and spatial resolution of SiPM detectors translate into sharper contrast, reduced noise, and greater confidence when interpreting subtle cortical hypometabolism in children. Such improvements are particularly relevant in a pediatric epilepsy context, where small foci of dysplasia or gliosis may be difficult to delineate, and patient motion can degrade image clarity [21,22]. By providing clearer metabolic maps, dPET has the potential to reduce equivocal findings that often necessitate invasive intracranial monitoring, thereby streamlining the presurgical workup and potentially shortening time to definitive surgical treatment [23]. Our 12-month Engel I seizure-freedom rate closely mirrors those reported in the pediatric epilepsy literature, suggesting that our overall surgical outcomes are comparable to established benchmarks [5]. However, given the limited size of our cohort, we are unable to draw statistically robust conclusions about the potential incremental impact of dPET versus aPET on postoperative seizure control.

Despite these encouraging results, several important limitations temper our conclusions. We excluded sedated patients, which may limit generalizability: in real-world pediatric PET imaging, sedation (e.g., midazolam, propofol) is often unavoidable and has been shown to modify cerebral glucose metabolism (e.g., propofol-induced global cortical suppression). Thus, our findings may not fully extend to sedated populations [24,25]. Furthermore, we did not perform direct, intra-patient comparisons of the two systems. Ethical and logistical constraints—chiefly concerns about additional radiation exposure and the challenges of sequential sedation or cooperation—precluded scanning the same child on both scanners. Instead, we compared two temporally separated cohorts: children imaged on an analog system in 2018–2019 and those scanned on a digital system in 2020–2021. As such, unmeasured differences in cohort characteristics—ranging from age distribution and epilepsy etiology to departmental workflow refinements—could contribute to the performance gap we observed.

Moreover, our retrospective, single-center design and limited sample size (*n* = 22) inherently reduce statistical power and generalizability. A study of this scale cannot reliably detect modest differences in detection rates, nor can it support granular subgroup analyses across specific pathologies or age brackets. Furthermore, our institutional limitations on pediatric anesthesia resulted in the exclusion of sedated or highly uncooperative children. This selection bias likely skewed our population toward older, more compliant patients, leaving unanswered questions about how dPET performs in very young or developmentally delayed children—precisely the patients who might benefit most from shorter acquisition times or motion-correction algorithms enabled by higher count statistics.

In addition to visual assessments, we included global quantitative z-score analyses based on a three-dimensional stereotactic surface projection database, which confirmed higher contrast with dPET. In this regard, more detailed voxel-wise receiver operating characteristic analyses, regional threshold optimizations, or even machine-learning–driven anomaly detectors could elucidate subtler distinctions between hardware platforms [26]. Artificial intelligence (AI) and automated classifiers are becoming increasingly important for the accurate categorization of neuroimaging data, with promising applications in the field of neurodegenerative diseases and growing potential in the assessment and management of epilepsy [27,28]. In cases where visual interpretation and global z-scores diverge, such advanced metrics might uncover hardware-driven performance nuances or inform future software enhancements.

It is worth mentioning the potential of reducing radiation dose or shortening the scan duration offered by dPET. Although our study maintained equivalent tracer activities and acquisition times across cohorts, the literature in adult oncology suggests that SiPM detectors can preserve diagnostic quality at 25–50% lower doses or with faster protocols [29]. Given the ALARA (as low as reasonably achievable) principle in pediatric imaging, prospective dose-optimization studies tailored to brain PET could yield significant safety benefits without sacrificing diagnostic efficacy. Along this path, newly developed dedicated brain PET scanners, such as the NeuroExplorer, may prove valuable for obtaining highly detailed images of deep brain structures, enabling the implementation of ultrafast imaging protocols [30].

In our MRI-negative cohort, PET successfully localized the epileptogenic zone in both aPET and dPET cases, underscoring its diagnostic utility. This finding reinforces the pivotal role of functional imaging for precise localization when MRI is unremarkable, and suggests that a multimodal approach can further improve seizure-freedom rates [13]. An additional consideration is the potential advantage of hybrid PET/MRI in delineating the epileptogenic focus. In a prospective ^18^F-FDG study on 31 patients, PET/MRI achieved a sensitivity for EZ detection of 77.4–90.3%, significantly outperforming PET/CT (58.1–64.5%) and standalone MRI (45.2–80.6%) (*p* < 0.0001) [16]. Moreover, visual lesion-boundary scores were higher with PET/MRI (2.8 ± 1.2) than with PET/CT (2.0 ± 1.1) or MRI alone (2.1 ± 1.2), representing a 51.9% increase in reader confidence over PET/CT. A separate pilot study in drug-resistant epilepsy demonstrated equivalent overall diagnostic accuracy for PET/MRI and PET/CT (87% vs. 85%, respectively), with comparable inter-reader agreement (kappa ≈ 0.8) and strong correlation in regional standardized uptake values (r = 0.99) [31]. However, it is important to note that first-generation PET/MRI platforms relied on magnetically shielded PMTs or MR-compatible avalanche photodiodes rather than SiPMs. Transitioning to SiPM technology promises several advantages in this hybrid setting: SiPMs are inherently immune to the MRI’s strong B-field, offer sub−300 ps timing resolution for enhanced time-of-flight performance, and enable a more compact, fully digital detector ring. Early prototype SiPM-based PET/MRI scanners have already shown improved energy and spatial resolution without compromising MR image quality [32,33]. Despite these clear benefits—seamless co-registration of molecular and structural data, elimination of ionizing X-ray dose from CT, and superior soft-tissue contrast—clinical adoption of PET/MRI remains limited by high capital and maintenance costs, along with the ongoing need for robust MR-based attenuation-correction algorithms and streamlined hybrid workflows [34].

Finally, even if dPET proves superior in image quality and reader confidence, real-world implementation hinges on cost and workflow factors. SiPM-based systems require considerable capital investment, additional maintenance, and staff training to develop and validate optimized pediatric protocols [35]. A thorough health-economic analysis is essential to weigh upfront expenditures against downstream savings achieved through reduced need for invasive monitoring, fewer repeat studies, and potentially improved surgical outcomes. Only by integrating performance metrics with cost–benefit evaluations can institutions make informed decisions about adopting this technology.

Further considerations include the impact of epilepsy etiology and concurrent antiseizure medications on imaging outcomes. Although hippocampal sclerosis and reactive gliosis often yield deeper and more extensive hypometabolic regions than focal cortical dysplasia, our modest, evenly matched cohorts (HS *n* = 10; FCD *n* = 5; gliosis *n* = 7) did not permit meaningful subgroup analyses of etiology-specific metabolic severity [36]. Likewise, all participants met drug-resistant epilepsy criteria; given that GABAergic agents (e.g., benzodiazepines, vigabatrin) can reduce cortical ^18^F-FDG uptake, a formal evaluation of how individual anti-seizure medication classes affect PET sensitivity fell outside this study’s primary aim of comparing real-world performance between analog and digital PET systems [37].

Taken together, our findings offer preliminary evidence that SiPM–based digital PET/CT enhances image clarity and reader consistency in pediatric epilepsy presurgical workups. Nonetheless, the absence of intra-patient comparisons and the modest, retrospective cohort design underscore the need for larger, prospective, multicenter studies. Future directions should include harnessing emerging AI-based reconstruction frameworks—such as deep convolutional neural networks with learned priors—which can boost PET spatial resolution and lesion contrast while enabling 30–50% reductions in scan time or injected activity [38,39]. Embedding these algorithms into simultaneous PET/MRI protocols allows MR-derived attenuation maps and segmentations to further refine reconstructions, achieving sub−5 mm effective resolution and flawless metabolic–anatomic co-registration [40]. Additionally, supervised machine-learning models trained on multimodal datasets can generate voxel-wise probability maps of hypometabolic foci, paving the way for automated, operator-independent EZ delineation. Collectively, these innovations promise faster, lower-dose examinations and more reproducible focus localization in pediatric presurgical planning.

Going forward, investigations should incorporate multicenter, randomized trials to increase both sample size and generalizability, and should specifically assess the benefits of digital PET’s rapid acquisition in younger or sedated children. In parallel, prospective cost–effectiveness and radiation dose–reduction analyses are needed to quantify the economic and safety advantages of dPET over analog systems. Finally, integrating AI-driven reconstruction algorithms and hybrid PET/MRI platforms holds promise for automated, high-resolution detection of hypometabolic foci. Addressing these avenues will allow future work to confirm and extend our preliminary findings on the clinical utility of digital PET in presurgical planning for pediatric epilepsy.

## 5. Conclusions

In this preliminary study, dPET yielded notably higher image quality and inter-observer agreement than analog PET/CT in pediatric epilepsy presurgical evaluation. Although EZ detection improvement lacked statistical significance in our small, retrospective cohorts, the enhanced clarity and consistency suggest meaningful clinical promise. Further prospective, intra-patient studies are needed to confirm these findings and assess their impact on surgical outcomes.

## Figures and Tables

**Table 1:** Patient clinical-demographic characteristics, PET and histological results.

Patient	Age	Sex	Epilepsy Duration (Years)	PET Device	PET Detection	EZ Location	MRI Findings	Histopathology	Engel Scale
1	11	F	2	aPET	+	R temp	−	Gliosis	Class I
2	13	M	3	aPET	+	L temp	+/C	HS	Class I
3 *	17	M	6	aPET	+	R temp-occip	+/C	Gliosis	Class I
4	8	M	2	aPET	−	L front	+	FCD	Class III
5	11	F	3	aPET	−	Left temp	+	HS	Class I
6	10	M	4	aPET	+	R temp	−	Gliosis	Class II
7	9	M	2	aPET	−	L front	+	FCD	Class I
8	15	M	3	aPET	+	L temp	+/C	HS	Class II
9	13	M	4	aPET	+	L temp	+/C	Temp cyst	Class I
10	9	F	2	aPET	+	R temp	−	HS	Class I
11	12	F	3	aPET	+	L front	+/C	FCD	Class II
12	15	M	4	dPET	+	R temp	+/C	HS	Class I
13	7	F	2	dPET	+	L front	+/C	FCD	Class I
14	9	M	3	dPET	+	R temp	−	HS	Class I
15	11	F	4	dPET	+	L temp	+/C	HS	Class I
16	13	F	5	dPET	+	R temp	−	HS	Class I
17	10	M	2	dPET	−	L front	+	FCD	Class I
18	16	M	3	dPET	+	R temp	+/C	HS	Class I
19	12	F	3	dPET	+	L temp	−	Gliosis	Class I
20	10	M	2	dPET	+	L temp	+/C	ODG WHO 2	Class III
21	9	M	3	dPET	+	R temp	+/C	HS	Class II
22	12	F	4	dPET	+	L front-temp	−	Gliosis	Class I

* Previously resected ependymoma; HS: hippocampal sclerosis; FCD: focal cortical dysplasia; temp: temporal; front: frontal; occip: occipital; aPET: analog PET; dPET: digital PET; ODG: oligodendroglioma; WHO: World Health Organization grade; L: Left; R: right; EZ: epileptogenic zone; C: concordance for EZ location between MRI and PET.

**Table 2:** Visual scores obtained on aPET and dPET systems.

Device	Average Visual Scores			ICC	95% (CI)
	Observer 1	Observer 2	Observer 3		
aPET	2.1 ± 0.9	2.2 ± 1.1	2 ± 0.8	0.63	0.2–0.8
dPET	3.1 ± 0.8	3 ± 1	3.1 ± 0.8	0.91	0.7–0.9

ICC: intraclass correlation coefficient; CI: confidence interval.

## Data Availability

The original contributions presented in this study are included in the article. Further inquiries can be directed to the corresponding author(s).

## Supplementary Materials

The following supporting information can be downloaded at https://www.mdpi.com/article/10.3390/biomedicines13081887/s1.
