# Supplementary material for: Clinical Performance of Analog and Digital 18F-FDG PET/CT in Pediatric Epileptogenic Zone Localization: Preliminary Results
Source: Biomedicines. 2025 Aug 3;13(8):1887. doi: 10.3390/biomedicines13081887 (PMC12383724; doi:10.3390/biomedicines13081887)
Supplement: Supplementary file 1 [file biomedicines-13-01887-s001.zip › biomedicines-3772142-supplementary.pdf]

Supplemental data S1 (analog and digital PET acquisition and reconstruction protocols)

Analog PET system

The PET-CT device was a Discovery ST (GE Healthcare, Milwaukee, Wisconsin, USA) with bismuth germanate crystal units arranged to form 24 rings combined with a 16-slice Light Speed Plus CT scanner. The average FWHM axial resolution of PET (full-width at half-maximum) was 5.2 mm and the system sensitivity was 9.3 cps/KBq for the 3D acquisition mode. PET/CT acquisition was started 60 min after 18F-FDG injection and lasted 10 min in all the subjects. Images were reconstructed using an ordered subset expectation maximization iterative algorithm (OSEM-SV, VUEPoint HD; GE Healthcare, 2iterations, 30 subsets), matrix 256 × 256; full width at half maxi-mum: 5 mm.

Digital PET system

Digital PET was carried out with Biograph Vision PET/CT system (Siemens Healthcare; Erlangen, Germany). A CT scan of the skull was performed with slice thickness of 1.0 mm, pitch factor 1, bone and soft tissue reconstruction kernels and maximum of 120 keV and 90 mAs by applying CARE kV and CARE Dose. After CT scanning, brain PET was acquired at 60 min post tracer administration in 3D (matrix: 440 × 440) with a zoom factor of 1.0. Images were reconstructed with a TrueX + TOF algorithm and Gauss-filtered to a transaxial resolution of 2 mm at FWHM (full width at half maximum). Attenuation correction was performed using the low dose non-enhanced computed tomography data.

Supplemental figure S1 (CortexID)

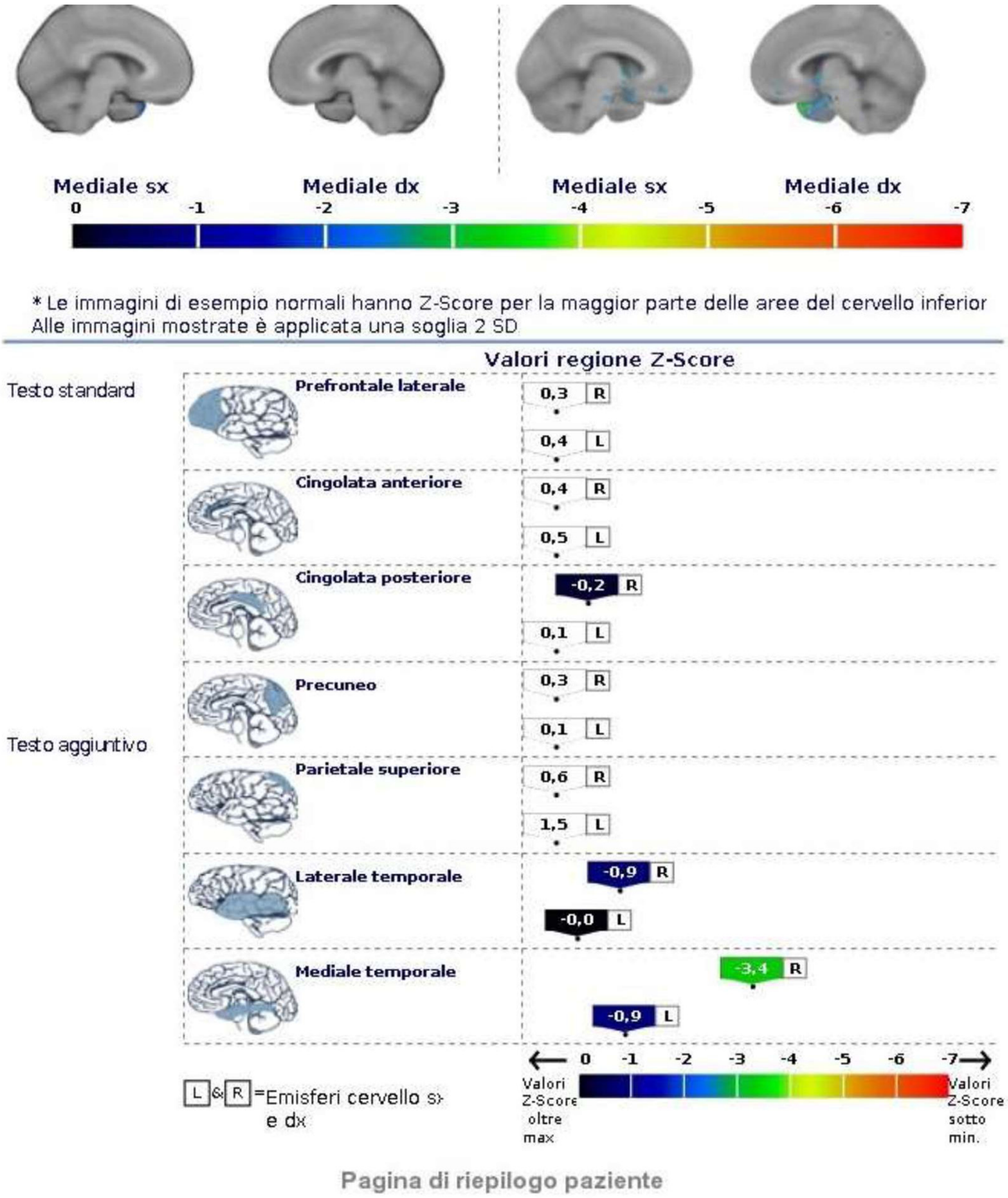

CortexID analysis of a 13-year old female patient with EZ located to the right temporal lobe, where quantitative evaluation identifies a hypometabolic focus with a z-score of -3.4 (text of the figure is in Italian).
